# A Hidden Markov Model to estimate population mixture and allelic copy-numbers in cancers using Affymetrix SNP arrays

**DOI:** 10.1186/1471-2105-8-434

**Published:** 2007-11-09

**Authors:** Philippe Lamy, Claus L Andersen, Lars Dyrskjot, Niels Torring, Carsten Wiuf

**Affiliations:** 1 Bioinformatics Research Center, University of Aarhus, Hoegh-Guldbergsgade 10, Bldg 1090, 8000 Aarhus C, Denmark; 2 Molecular Diagnostic Laboratory, Aarhus University Hospital, Skejby, Brendstrupgaardsvej 100, 8200 Aarhus N, Denmark

## Abstract

**Background:**

Affymetrix SNP arrays can interrogate thousands of SNPs at the same time. This allows us to look at the genomic content of cancer cells and to investigate the underlying events leading to cancer. Genomic copy-numbers are today routinely derived from SNP array data, but the proposed algorithms for this task most often disregard the genotype information available from germline cells in paired germline-tumour samples. Including this information may deepen our understanding of the "true" biological situation e.g. by enabling analysis of allele specific copy-numbers. Here we rely on matched germline-tumour samples and have developed a Hidden Markov Model (HMM) to estimate allelic copy-number changes in tumour cells. Further with this approach we are able to estimate the proportion of normal cells in the tumour (mixture proportion).

**Results:**

We show that our method is able to recover the underlying copy-number changes in simulated data sets with high accuracy (above 97.71%). Moreover, although the known copy-numbers could be well recovered in simulated cancer samples with more than 70% cancer cells (and less than 30% normal cells), we demonstrate that including the mixture proportion in the HMM increases the accuracy of the method. Finally, the method is tested on HapMap samples and on bladder and prostate cancer samples.

**Conclusion:**

The HMM method developed here uses the genotype calls of germline DNA and the allelic SNP intensities from the tumour DNA to estimate allelic copy-numbers (including changes) in the tumour. It differentiates between different events like uniparental disomy and allelic imbalances. Moreover, the HMM can estimate the mixture proportion, and thus inform about the purity of the tumour sample.

## Background

Chromosomal abnormalities such as loss-of-heterozygosity (LOH) or genomic copy-number changes are frequent in tumour cells. LOH occurs when a heterozygous marker in germline DNA of an individual becomes homozygous in cancer DNA of the same individual. This event is the result of losing one allele of a chromosomal region while the other allele is retained, duplicated (uniparental disonomy), or multiplicated (uniparental polysomy). In the same way, chromosomal amplifications can be unbalanced (if only one allele of a chromosomal region is multiplicated) or balanced (if both alleles are multiplicated). Detecting chromosomal abnormalities is important in cancer research as it allows the discovery of chromosomal regions possibly harbouring cancer-related genes such as tumour suppressor genes or oncogenes. It may also be used to identify genomic markers (i.e. chromosomal abnormalities) that may distinguish between clinically important stages in the disease course, e.g. markers of metastasis or markers of treatment response.

Single nucleotide polymorphisms (SNPs) account for most of the genetic variation in the human genome. They occur every 100 to 300 bases along the 3-billion-base human genome [1]. Different techniques (e.g. Illumina [2], Affymetrix [3], Perlegen [4]) have been developed in order to genotype thousands of SNPs distributed all over the genome at the same time. In this paper, we focus on Affymetrix SNP-arrays, but note that the method we have developed can be applied to data obtained from other experimental platforms as well.

The Affymetrix technique is based on genomic hybridization to synthetic high-density oligonucleotide microarrays. Each of the two alleles of a SNP is represented by 10 oligonucleotides (together called a probeset) and hybridization (probe) intensities are measured for all probes in the probeset [3]. Different algorithms [5-8], have been developed to genotype correctly SNPs from the Affymetrix intensities. A very high accuracy and concordance of genotype calls is observed for normal samples as the ploidy is always two. However, it is much more diffcult to genotype cancer samples due to genomic alterations that might change the ploidy number.

Hidden Markov Models (HMMs) have been used extensively to recover unobserved underlying states that give rise to an observed sequence of data. In relation to LOH analyses HMMs have been used to infer whether an allele is lost or retained (i.e. two hidden states) from genotype data [9-11]. Lin et al. [10] and Koed et al. [9] developed HMM methods that score the presence of allelic imbalance mainly based on converted SNPs (when AB call becomes AA or BB in the cancer sample). In [11], Beroukhim et al. describes a HMM-based method to identify LOH from unpaired tumour samples. They use the genotype calls to identify whether a SNP marker is in a retention state or in a LOH state. By integrating copy-number analysis into the analysis, they can distinguish LOH from allelic imbalance. However, the LOH analysis and the copy-number analysis are performed separately. Besides, the LOH analysis is highly dependent on the genotype calls even if the possibility of genotyping errors is taken into account.

HMMs have also been used for copy-number analysis. In [12], Fridlyand et al. developed a HMM to analyse microarray-based comparative genomic hybridization (array CGH) data. In [13], Zhao et al. developed a method to infer DNA copy-numbers using Affymetrix SNP-arrays. They combined probeset intensities for each SNP into a single value and used the values as an observed sequence of data in their HMM. These methods are not allele specific and thus cannot distinguish e.g. retention (keeping both alleles) from uniparental disomy (losing one allele and duplicating the other one), which appears to be very important and wide-spread in certain cancers [14].

More recently, methods to infer allele specific copy-numbers have been published [15,16]. Laframboise et al. [15] used a circular binary segmentation (CBS) algorithm which originally was used for array CGH [17]. Huang et al. [16] used a kernel smoothing method to estimate allelic copy-number changes. In [18], Nannya et al. describes a HMM to infer allelic copy-numbers that is based on the observed sequence of SNPs intensities ratios for which the corresponding normal SNP markers are heterozygous.

In this study, we developed a HMM method to infer allele specific copy-numbers using Affymetrix SNP arrays. In a sense the method works on paired normal-tumour samples. It takes as input the genotype calls of the normal sample, the allelic specific intensities of the tumour sample and outputs the estimated copy-number states of each allele for each SNP. To limit the state space of the underlying Markov Chain, we restricted the possible copy-numbers of each allele to 0, 1, 2 and > 2. Many tumour samples contain a large fraction of normal cells and this potentially affects the performance of the method. We therefore included the possibility to estimate the population mixture (proportion of cancer cells; henceforth called mixture proportion) from the data and used this in the analysis. We did this in a way similar to Fridlyand et al.'s method for array CGH [12]. We tested our HMM model on simulated data sets, normal samples from the HapMap project and on bladder and prostate tumour samples.

## Results and Discussion

We first normalized the 90 HapMap arrays and the 134 cancer arrays and transformed the allele intensities as described in Methods. We then selected SNPs for each of the three groups of arrays: the HapMap, bladder and prostate groups. The selection was done using only the normal samples from each group as described in Methods. After selection, we had 17,198 SNPs selected for the HapMap group, 15,237 SNPs for the bladder group and 17,541 SNPs for the prostate group.

**Figure 1:**
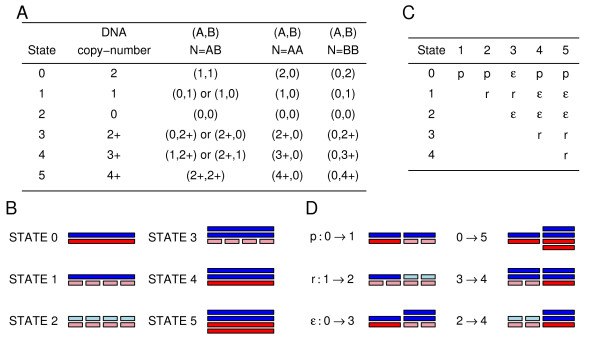
**States and transition matrix of the HMM**. **A**. This figure shows the definition of the states in the HMM. The genotype call for the germline DNA is given by the letter N = AB, AA or BB. For each state, the total DNA copy-number and the allelic copy-numbers are given. State 0 is the germline state also called the normal state; state 1 corresponds to a heterozygous deletion (loss of one allele); state 2 corresponds to a homozygous deletion (loss of two alleles); state 3 corresponds to uniparental di/polysomy (loss of one allele and duplication or multiplication of the other allele); state 4 corresponds to unbalanced amplification (duplication or multiplication of only one allele); state 5 corresponds to balanced amplification (duplication or multiplication of both alleles). Notice that when the SNP marker in the germline DNA is homozygous, states 3, 4 and 5 are very similar and states 0 and 3 cannot be differentiated in case of uniparental disomy. **B**. Visual interpretation of the states. **C**. Transition matrix. The transition probabilities are the probabilities to move from one state for a SNP to another state for the next SNP. The rest of the matrix is given by the detailed balance equation and symmetry. **D**. Visual interpretation of the transition parameters. The figure represents two consecutive SNPs in the sample.

The normalized allele intensities and the genotypes of the germline DNA were used as input in our HMM. The HMM outputs for each selected SNP the allelic copy-number. To limit the number of states of the HMM, we defined six categories corresponding to different events: germline state or normal state, heterozygous deletion state, homozygous deletion state, uniparental disomy or uniparental polysomy state, unbalanced amplification state and balanced amplification state (Figures 1A and 1B). The transition probabilities (probabilities to move from one state to another when considering two consecutive SNPs) are defined using three parameters: two variable parameters, *p *and *r *and one fixed parameter, *ε *(see Methods and Figures 1C and 1D). The *p *parameter corresponds to jumping from the normal state to an abnormal state, the *r *parameter corresponds to jumping between two abnormal states and the *ε *parameter corresponds to jumping between two states involving double events. Here *ε *is fixed to 0.00001.

### Estimating the parameters

We simulated data sets with known transition parameters and investigated how the true parameters and true states were recovered. Six samples were created for each combination of transition parameters. The parameters varied between 0.001 and 0.1. A value of 0.001 means that a change of states occurs every 1000 SNPs on average. We observed that our method is able to recover the true values of the transition parameters with a good accuracy. There is however a tendency to slightly underestimate the parameters when they are high. Moreover, the method is able to recover the hidden states with a very good accuracy (from 97.71% to 99.97%). The worst case is seen when both transition parameters are high, i.e., when there are many copy-number changes (Table 1).

**Table 1:** Estimation the transition parameters and the states

		True *r*			
True *p*	Estimation	0.001	0.01	0.05	0.1
	*p*	0.00115	0.00104	0.00101	0.00107
0.001	*r*	0.00108	0.01028	0.04889	0.09462
	Accuracy	0.99965	0.99893	0.99487	0.99227

	*p*	0.00959	0.00994	0.00934	0.01006
0.01	*r*	0.00112	0.01057	0.04931	0.09588
	Accuracy	0.99725	0.99622	0.99303	0.98850

	*p*	0.04861	0.04831	0.04794	0.04849
0.05	*r*	0.00109	0.01034	0.04812	0.09402
	Accuracy	0.99180	0.99000	0.98517	0.98018

	*p*	0.09551	0.09356	0.09603	0.09300
0.1	*r*	0.00140	0.01006	0.04857	0.09480
	Accuracy	0.99233	0.98980	0.98269	0.97708

For each pair of true *p *and *r *parameters, we simulated six samples where all SNPs were given a heterozygous call in the germline DNA. Here we report the average of the estimation for *p *and *r *and how often the true copy-number state is recovered.

**Figure 2:**
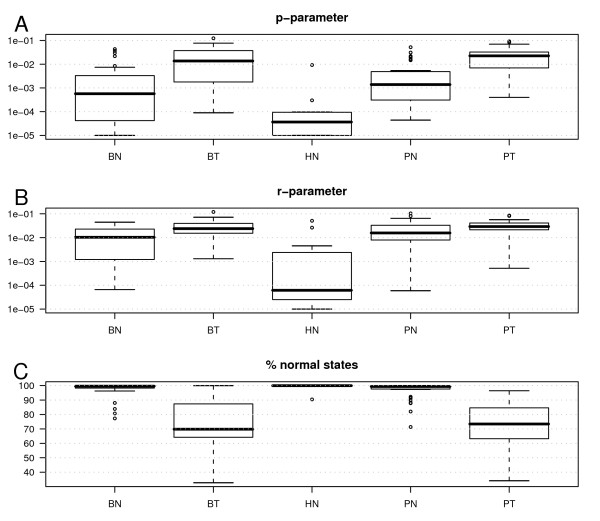
**Estimation of the transition parameters and percentage of SNPs in state 0 in the real data**. **A**. Boxplots for the *p*-parameter. **B**. Boxplots for the *r*-parameter. **C**. Boxplots for the percentage of estimated SNPs in state 0 (normal state). BN: Bladder Normal samples; HN: Hapmap Normal samples; PN: Prostate Normal samples; BT: Bladder Tumour samples; PT: Prostate Tumour samples.

**Figure 3:**
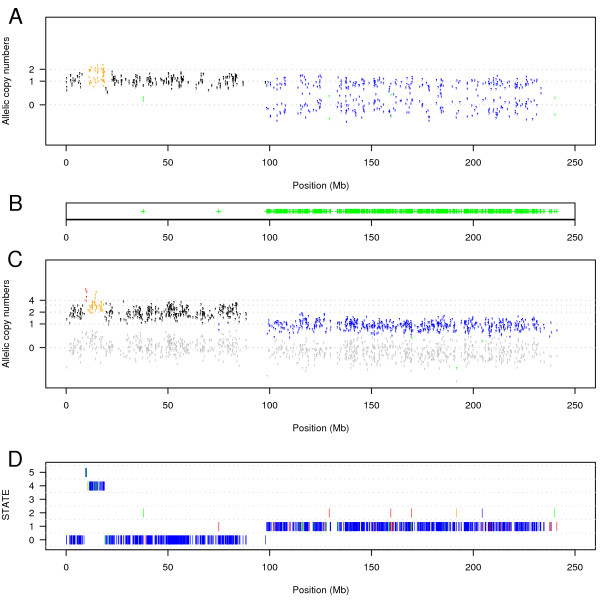
**Chromosome 2 in a bladder tumour sample**. In this chromosome, we can distinguish two events: an unbalanced amplification coloured in orange (only one allele is duplicated) and a heterozygous deletion of the q-arm coloured in blue. **A**. For each SNP heterozygous in the germline DNA, the normalized intensities (as defined in Methods equation 4) of each allele are plotted. The colours represent the estimated state of the SNP: black for state 0 (germline state), blue for state 1 (heterozygous deletion: loss of one allele), green for state 2 (homozygous deletion: loss of both alleles), purple for state 3 (uniparental di/polysomy: loss of one allele and multiplication of the other one), orange for state 4 (unbalanced amplification: multiplication of one allele) and red for state 5 (balanced amplification: multiplication of the two alleles). **B**. Shown is the region of LOH. **C**. For each SNP homozygous in the germline DNA, the normalized intensities (as defined in Methods equation 4) of each allele are plotted. The absent allele is coloured in grey. **D**. Shown is the estimated sequence of hidden states. The colours indicate the posterior probabilities of the states: blue > 0.99, green > 0.95, orange > 0.9 and red < 0.9.

Next, we applied the method to 18 HapMap samples and to the bladder and prostate samples (Figure 2). The transition parameters which are assumed to be the same for all chromosomes in a sample were estimated for each sample. The results show that the estimated transition parameters are within the range where the method obtains a very good accuracy in the simulated data sets. The median for the estimated *p *is 0.00036 in the normal samples and 0.02188 for the tumour samples. Similarly, the median is 0.00956 for the estimated *r *in the normal samples and 0.02764 for the tumour samples. Besides, the average percentage of SNPs being in normal state (state 0) was 99.44 for the HapMap samples, 96.93 for the bladder normal samples and 96.57 for the prostate normal samples. Moreover, the normal samples with the lowest percentages of SNPs in state 0 are the ones flagged as bad arrays (high percentage of outliers) by the dChip software. Three normal samples from the bladder group were flagged as bad arrays. Figure 3 shows the results of our method on one bladder cancer sample.

### Accuracy of the method when the sample is a population mixture

**Figure 4:**
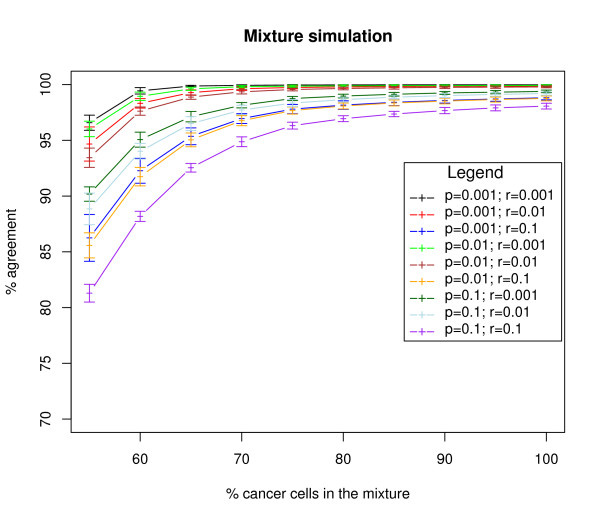
**Accuracy of our method on simulated data**. The percentage of agreement between the recovered state and the original state in the simulated data sets is plotted as a function of the population mixture (percentage of tumour cells in the sample). The simulation were done using different combinations of transition parameters.

Using different values for the transition parameters, we simulated data sets where the sample was a mixture of normal cells and cancer cells. The percentage of cancer cells was chosen between 55% and 100%. The accuracy of the method was estimated assuming the sample was not a mixture (Figure 4). It is observed that if the mixture level is over 70%, then the true hidden states are recovered with high accuracy (above 94%).

In the simulated data, amplification of an allele always implies that the copy-number increases by one; e.g. if the SNP is heterozygous then amplification of the A allele results in two A alleles. In real data, this is not always true: amplification may increase the allelic copy-number by more than one. Thus it is easier for the method to recover the true hidden states in the simulated data than in real data.

### Estimating the population mixture

We simulated data sets based on the results from the analysis of the bladder and prostate tumours. Using the estimated hidden states, data sets with known population mixture were created by adding a percentage of normal cells. Subsequently the mixture proportion was estimated. This was done on the 29 bladder samples for different mixture levels (60, 70, 80 and 90%) (Table 2).

**Table 2:** Estimation of the mixture proportion

	Estimated mixture		Accuracy (%)	
True mixture	Average	Stdev	Without mixture	With mixture
0.60	0.603	0.012	90.31	95.45
0.70	0.719	0.069	94.43	96.16
0.80	0.803	0.009	96.87	97.52
0.90	0.902	0.014	98.16	98.25

Simulated data sets were created based on the analyses of the bladder tumour samples. Here the average is calculated for all samples where the amount of informative SNPs (states 1 to 5) was suffciently high (> 5%, see Methods). The number of correctly identified states is shown with and without estimation of the mixture proportion.

Reliable information about mixture is only available if the sample contains SNPs with different copy-number alterations. For example, if the observed copy-number of a SNP is 4.7, then it is not possible to distinguish between a mixture of 1) 90% tumour cells with 5 copies and 10% normal cells (2 copies) and 2) 54% tumour cells with 7 copies and 46% normal cells. However, if SNPs exist in several different states, then it becomes possible to distinguish between different mixtures. In case 1), a SNP in state 1 will have an observed copy-number of 1.1 and in case 2), the observed copy-number will be 1.46.

Knowledge of the mixture level helps to get a more accurate recovery of the hidden states. When the mixture level is about 60%, the accuracy rises from about 90% to 95% (Table 2). We also estimated the mixture proportion on the real bladder and prostate data sets. As all the prostate samples were microdissected and all the bladder samples were macrodissected, we expected the samples to be almost pure cancer cells (Table 3). We observed that most of our samples showed no evidence of being a mixture. However, 6 bladder samples (out of 18) and 4 prostate samples (out of 25) presented some mixture level. As all samples were microdissected or macrodissected, the estimated mixture level may not reflect a true mixture of cancer/normal cells. Instead it may reflect heterogeneity in the cancer cells which is supported by findings in the literature. In bladder cancer, cells with different genomic alterations have been found in the same tumour [19]. In prostate cancer, genomic heterogeneity has been reported in several papers; e.g. [20,21]. However, it is not straightforward to modify the HMM to operate on mixtures of cancer cells.

**Table 3:** Estimation of the mixture proportion (*m*) in the bladder and the prostate groups

			*M *< 1	
Cancer type	Number of samples	Number of samples with *m *= 1	Average	Stdev
Bladder	18	12	0.793	0.193
Prostate	25	21	0.93	0.055

For all bladder and prostate tumour samples, the mixture proportion was estimated. The averages and standard deviations are only calculated for the tumour samples whose estimates of mixture proportion, *m*, were not 1 (100% cancer cells).

### Varying the transition parameters across the chromosomes

Until now, the transition parameters were estimated for each sample and were chromosome independent. However, it is known that for a given cancer type certain chromosomes are more prone to abnormalities than others. In order to take this into account, we simulated 40 samples where the transition parameters differed for each chromosome, but were similar for each sample (see Methods). The transition parameters were randomly chosen between 0.001 and 0.05. We then analysed the samples and estimated the accuracy of the method (Table 4). The simulated samples were divided into two categories: one where the germline genotype calls are only heterozygous and one where the germline genotype calls have the same distribution as in a normal sample (30% of the SNP markers are heterozygous). This partition of the samples showed that the addition of homozygous SNP markers decreased the accuracy of the method slightly (from 99.50% to 98.55%).

**Table 4:** Compararison of two estimation methods on 40 simulated samples

		Accuracy in %		Average posterior probability	
Method	Number of samples	Average	stdev	True state	False state
One-array					
- only normal heterozygous calls	20	99.495	0.058	0.997	0.812
- all calls	20	98.548	0.147	0.992	0.845
All-array					
- only normal heterozygous calls	20	99.552	0.042	0.997	0.806
- all calls	20	98.653	0.150	0.992	0.830

Here the one-array method (transition parameters are estimated for each sample and are the same for all chromosomes) and the all-array method (transition parameters are the same for each chromosome across all samples) were compared. For each method, the accuracy of the HMM on samples where all SNPs are heterozygous in the normal sample and the accuracy of the HMM on samples where the call distribution is the same as a HapMap sample (all calls) were also compared. Moreover, an average of the posterior probability for all SNPs correctly recovered or not is given.

In order to account for the similarities between different samples from the same cancer type, we also estimated the transition parameters for each chromosome across all samples of a group. This modified version allows the chromosomes to be ranked according to the frequency of changes occurring as reflected in the estimated transition parameters. We ran the modified version on the same 40 simulated samples (Table 4). As expected, we achieved a slightly better accuracy in recovering the hidden states. The two methods agreed on 99.70% of the recovered states when we put no restrictions on the genotypes and on 99.86% of the recovered states when all SNPs were assumed to be heterozygous.

We applied our all-array method to the set of bladder and prostate tumours and compared the results obtained analysing one sample at a time. The two methods agreed on 95.71% of the states for the bladder group and on 96.24% of the states for the prostate group. From the results of the all-array method, we were also able to classify chromosomes according to how often a change in copy-number occurs. For the bladder group, copy-number changes occurred most often in chromosomes 8 and 9. These two chromosomes are known to be frequently abnormal in bladder tumours [22,23]. For the prostate group, copy-number changes occurred most often in chromosomes 3, 7, 8 and 16. A combined analysis of published CGH studies [24] and a study based on SNP arrays [25] showed that these chromosomes are frequently abnormal in prostate tumours.

### Uniparental disomy

**Figure 5:**
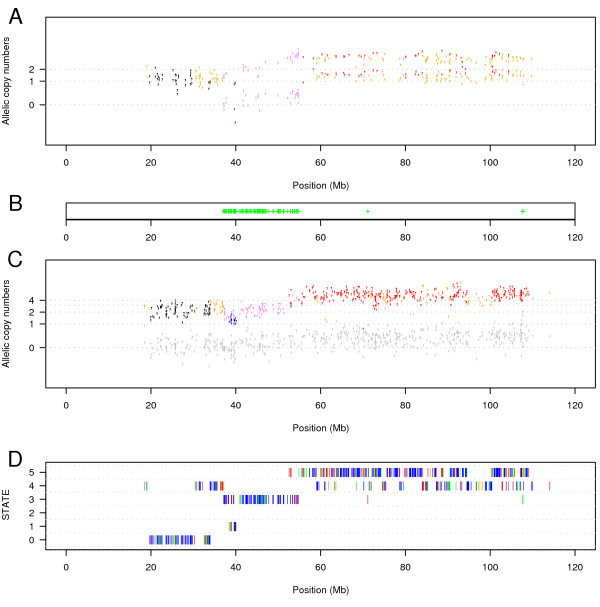
**An example of uniparental disomy in chromosome 13 in a bladder tumour sample**. In this chromosome, we can distinguish uniparental disomy coloured in purple in a region of approximatively 20 Mb and an unbalance amplification in the rest of the q-arm coloured in orange and red. **A**. For each SNP heterozygous in the germline DNA, the normalized intensities (as defined in Methods equation 4) of each allele are plotted. The colours represent the estimated state of the SNP: black for state 0, blue for state 1, green for state 2, purple for state 3, orange for state 4 and red for state 5. **B**. Shown is the region of LOH. **C**. For each SNP homozygous in the germline DNA, the normalized intensities (as defined in Methods equation 4) of each allele are plotted. The absent allele is coloured in grey. **D**. Shown is the estimated sequence of hidden states. The colour indicates the posterior probabilities of the states: blue > 0.99, green > 0.95, orange > 0.9 and red < 0.9.

Uniparental disomy occurs when one allele of a chromosomal region is lost and the remaining allele is duplicated. In a sample this means that SNPs in such a region will lose their heterozygosity while the copy-number will remain normal (2 copies) or higher. Andersen et al. [14] and Raghavan et al. [26] showed that uniparental disomy is frequent in colorectal cancer and in acute myeloid leukemias, respectively. In bladder and prostate cancer, we also found some examples of uniparental disomy. Figure 5 shows an example of uniparental disomy in chromosome 13 of a bladder sample, demonstrating that the HMM successfully can find cases of uniparental disomy.

### Using the homozygous SNPs to estimate allelic copy-number changes

In our HMM approach, there are two ways to estimate allelic copy-number changes. One can choose to use only the SNPs which are heterozygous in the germline sample or to use all SNPs including the homozygous SNPs. Both ways have been tested here. Using only the SNPs which are heterozygous in the germline sample is the best way to obtain good estimates of the underlying states because all states of the HMM are differentiable. In this paper, we obtained a high rate of recovery (above 99.40%, Table 4) when the simulated samples had only heterozygous calls. However, the average heterozygosity in the Affymetrix Genechip 100 k SNP arrays is only around 0.3 [27]. This implies that less than one third of the SNPs are heterozygous in a normal sample. Therefore, including SNPs with homozygous calls in the germline sample may improve the resolution of the map of allelic copy-number changes. When we included all genotype calls in the analysis, we still obtained a high rate of recovery (above 98.50%, Table 4). SNPs with homozygous genotypes in the germline sample can also differentiate different states based on their different copy-numbers. However, some states are very similar: states 0 and 3 or states 3, 4 and 5. The presence of heterozygous SNPs helps in differentiating these states. Oppositely the presence of homozygous SNPs might help in differentiating between heterozygous states (e.g. states 0 and 4; see Figure 1); for example, if noise is corrupting the signal from a heterozygous SNP with homozygous neighbours, then the copy numbers of the neighbours can point to whether the heterozygous SNP is in state 0 or 4.

### Comparison with PLASQ

PLASQ [15] was run on 10 of the real samples (6 prostate and 4 bladder samples). The states estimated by PLASQ were converted into the corresponding states in our model and the results were compared. Agreement between the two methods was on average 90.47% (ranging from 77.05% to 98.11%). Generally, our method detects more abnormalities than PLASQ. This is concordant with previous observations concerning PLASQ, where it has been found that PLASQ is conservative [28]; i.e. PLASQ has a tendency to prefer the normal state. In order to be more conservative, we ran our HMM with a higher standard deviation in the emission density for the normal state. As expected, agreement between the two methods increased to an average of 95.02% (ranging from 88.86% to 99.38%). Additionally we calculated the average posterior probabilities of the states when both methods agree or disagree. As expected, when both methods agree, the average is higher than when they disagree (0.983 against 0.909).

## Conclusion

In this study, we described a HMM-based method to estimate allelic SNP copy-number changes, LOH and allelic imbalance using Affymetrix GeneChip SNP arrays. The method takes as input the genotype call of the germline sample and the allelic SNP intensities of the tumour sample and outputs the estimated copy-number states for each SNP. The different hidden states estimated by the HMM correspond to different events occurring in the cancer cell. A chromosomal region may remain unchanged in a cancer cell, may lose one allele (LOH event) or both alleles (homozygous deletion), may lose one allele and multiplicate the other one (LOH+uniparental disomy), may multiplicate one allele (allelic imbalance) or both alleles (see Figure 1). Our method is able to reliably differentiate between these events.

When samples are taken from tumour tissue, they often contain a mixture of normal and cancer cells. Different techniques such as microdissection can help keep the percentage of normal cells low but this is a procedure that cannot be done automatically and is not always done. In this study, we showed that it is possible to estimate the true mixture proportion of a sample. We also showed that knowledge of the mixture proportion improves estimation of the allelic copy-numbers. In fact, the SNP intensity reflects the average copy-number of that particular SNP in the different cells in the sample. However, population mixture of normal and cancer cells might be confused with tumour heterogeneity. Multiclonality has been shown to occur in bladder cancer as well as in prostate cancer and this could also lead to non-integer copy-numbers, i.e. the average over all cells is not an integer. It would be interesting to tackle this issue in future work.

Finally, we discussed the utility of using SNPs which are homozygous in the germline samples in the estimation of allelic copy-number changes. We showed that despite the fact that they cannot really differentiate between events by themselves, e.g. normal state and uniparental disomy with a single duplication, they are useful in getting a finer map of copy-number changes in the cancer.

## Methods

### Materials

We used tumour and blood samples from 38 patients diagnosed with prostate cancer and 29 patients diagnosed with bladder cancer. All the bladder tumour samples were macrodissected. This implies that any connective tissue and muscle tissue were scraped away with a scalpel while looking at the tumour section in a microscope [29]. All the prostate samples were laser microdissected [30]. The GeneChipMapping 100 K array was applied to all samples. Only the array probes for Xba I cleaved DNA were used. We also downloaded the 100 K Affymetrix SNP arrays from the 30 CEPH trios (90 samples) used in the international Hapmap project [31]. Only the Xba arrays were used.

### Normalization and allele copy-numbers of SNPs

The probe set intensities of all arrays were normalized using the dChip software (Invariant Set ormalization) [10]. Subsequently, the intensities were combined into two values (intensities of A and B alleles) by taking the logarithm of the average over all Perfect Match (PM) probes for the *α *allele, *α *= A or B, i.e.

Ii(α)=log⁡(1p∑j=1pPMij(α)),
 MathType@MTEF@5@5@+=feaafiart1ev1aaatCvAUfKttLearuWrP9MDH5MBPbIqV92AaeXatLxBI9gBaebbnrfifHhDYfgasaacPC6xNi=xI8qiVKYPFjYdHaVhbbf9v8qqaqFr0xc9vqFj0dXdbba91qpepeI8k8fiI+fsY=rqGqVepae9pg0db9vqaiVgFr0xfr=xfr=xc9adbaqaaeGacaGaaiaabeqaaeqabiWaaaGcbaGaemysaK0aaSbaaSqaaiabdMgaPbqabaGccqGGOaakiiGacqWFXoqycqGGPaqkcqGH9aqpcyGGSbaBcqGGVbWBcqGGNbWzdaqadaqaaKqbaoaalaaabaGaeGymaedabaGaemiCaahaamaaqahabaGaemiuaaLaemyta00aaSbaaeaacqWGPbqAcqWGQbGAaeqaaiabcIcaOiab=f7aHjabcMcaPaqaaiabdQgaQjabg2da9iabigdaXaqaaiabdchaWbGaeyyeIuoaaOGaayjkaiaawMcaaiabcYcaSaaa@4C0A@

where *PM*_*ij*_(*α*) is the intensity of the *j*-th probe of allele *α *for SNP *i*. Here *j *runs over *j *= 1,...,*p*, where *p *= 10, *i *= 1,..., 57290. *p *is the number of probe in a probeset interrogating one allele and *i *is the total number of SNPs.

Based on the observation and model described in [8], we have for each SNP *i*:

log⁡(Mi2(α))=c1+c2log⁡(Mi1(α))
 MathType@MTEF@5@5@+=feaafiart1ev1aaatCvAUfKttLearuWrP9MDH5MBPbIqV92AaeXatLxBI9gBaebbnrfifHhDYfgasaacPC6xNi=xI8qiVKYPFjYdHaVhbbf9v8qqaqFr0xc9vqFj0dXdbba91qpepeI8k8fiI+fsY=rqGqVepae9pg0db9vqaiVgFr0xfr=xfr=xc9adbaqaaeGacaGaaiaabeqaaeqabiWaaaGcbaGagiiBaWMaei4Ba8Maei4zaCMaeiikaGIaemyta00aa0baaSqaaiabdMgaPbqaaiabikdaYaaakiabcIcaOGGaciab=f7aHjabcMcaPiabcMcaPiabg2da9iabdogaJnaaBaaaleaacqaIXaqmaeqaaOGaey4kaSIaem4yam2aaSbaaSqaaiabikdaYaqabaGccyGGSbaBcqGGVbWBcqGGNbWzcqGGOaakcqWGnbqtdaqhaaWcbaGaemyAaKgabaGaeGymaedaaOGaeiikaGIae8xSdeMaeiykaKIaeiykaKcaaa@4C85@

where *α *is allele A or B, Mi2(α)
 MathType@MTEF@5@5@+=feaafiart1ev1aaatCvAUfKttLearuWrP9MDH5MBPbIqV92AaeXatLxBI9gBaebbnrfifHhDYfgasaacPC6xNi=xH8viVGI8Gi=hEeeu0xXdbba9frFj0xb9qqpG0dXdb9aspeI8k8fiI+fsY=rqGqVepae9pg0db9vqaiVgFr0xfr=xfr=xc9adbaqaaeGacaGaaiaabeqaaeqabiWaaaGcbaGaemyta00aa0baaSqaaiabdMgaPbqaaiabikdaYaaakiabcIcaOGGaciab=f7aHjabcMcaPaaa@32D2@ is the mean intensity for allele *α *in samples with two copies of *α*, Mi1(α)
 MathType@MTEF@5@5@+=feaafiart1ev1aaatCvAUfKttLearuWrP9MDH5MBPbIqV92AaeXatLxBI9gBaebbnrfifHhDYfgasaacPC6xNi=xH8viVGI8Gi=hEeeu0xXdbba9frFj0xb9qqpG0dXdb9aspeI8k8fiI+fsY=rqGqVepae9pg0db9vqaiVgFr0xfr=xfr=xc9adbaqaaeGacaGaaiaabeqaaeqabiWaaaGcbaGaemyta00aa0baaSqaaiabdMgaPbqaaiabigdaXaaakiabcIcaOGGaciab=f7aHjabcMcaPaaa@32D0@ is the mean intensity for allele *α *in samples with one copy of *α *and *c*_1 _and *c*_2 _are SNP-independent parameters. (see additional file 1 as an illustration). Note that the means are SNP-dependent. Assuming that the logarithm of the copy-number of a SNP allele is proportional to the logarithm of its intensity, see e.g. [32], we have for *C*_*i*_(*α*) > 0:

log⁡2(Ci(α))=ai+bilog⁡2(Mic(α)),
 MathType@MTEF@5@5@+=feaafiart1ev1aaatCvAUfKttLearuWrP9MDH5MBPbIqV92AaeXatLxBI9gBaebbnrfifHhDYfgasaacPC6xNi=xI8qiVKYPFjYdHaVhbbf9v8qqaqFr0xc9vqFj0dXdbba91qpepeI8k8fiI+fsY=rqGqVepae9pg0db9vqaiVgFr0xfr=xfr=xc9adbaqaaeGacaGaaiaabeqaaeqabiWaaaGcbaGagiiBaWMaei4Ba8Maei4zaC2aaSbaaSqaaiabikdaYaqabaGccqGGOaakcqWGdbWqdaWgaaWcbaGaemyAaKgabeaakiabcIcaOGGaciab=f7aHjabcMcaPiabcMcaPiabg2da9iabdggaHnaaBaaaleaacqWGPbqAaeqaaOGaey4kaSIaemOyai2aaSbaaSqaaiabdMgaPbqabaGccyGGSbaBcqGGVbWBcqGGNbWzdaWgaaWcbaGaeGOmaidabeaakiabcIcaOiabd2eannaaDaaaleaacqWGPbqAaeaacqWGJbWyaaGccqGGOaakcqWFXoqycqGGPaqkcqGGPaqkcqGGSaalaaa@4FDB@

where *C*_*i*_(*α*) and Mic(α)
 MathType@MTEF@5@5@+=feaafiart1ev1aaatCvAUfKttLearuWrP9MDH5MBPbIqV92AaeXatLxBI9gBaebbnrfifHhDYfgasaacPC6xNi=xH8viVGI8Gi=hEeeu0xXdbba9frFj0xb9qqpG0dXdb9aspeI8k8fiI+fsY=rqGqVepae9pg0db9vqaiVgFr0xfr=xfr=xc9adbaqaaeGacaGaaiaabeqaaeqabiWaaaGcbaGaemyta00aa0baaSqaaiabdMgaPbqaaiabdogaJbaakiabcIcaOGGaciab=f7aHjabcMcaPaaa@332F@ are the copy-number and intensity of allele *α *in SNP *i*, respectively. The parameters *α*_*i *_and *b*_*i *_are SNP-specific. Here we allow *C*_*i*_(*α*) to be an arbitrary number to allow for mixed samples.

From equations 2 and 3, we derive *C*_*i*_(*α*), the allelic copy-number, given Mi1(α)
 MathType@MTEF@5@5@+=feaafiart1ev1aaatCvAUfKttLearuWrP9MDH5MBPbIqV92AaeXatLxBI9gBaebbnrfifHhDYfgasaacPC6xNi=xH8viVGI8Gi=hEeeu0xXdbba9frFj0xb9qqpG0dXdb9aspeI8k8fiI+fsY=rqGqVepae9pg0db9vqaiVgFr0xfr=xfr=xc9adbaqaaeGacaGaaiaabeqaaeqabiWaaaGcbaGaemyta00aa0baaSqaaiabdMgaPbqaaiabigdaXaaakiabcIcaOGGaciab=f7aHjabcMcaPaaa@32D0@ and Mic(α)
 MathType@MTEF@5@5@+=feaafiart1ev1aaatCvAUfKttLearuWrP9MDH5MBPbIqV92AaeXatLxBI9gBaebbnrfifHhDYfgasaacPC6xNi=xH8viVGI8Gi=hEeeu0xXdbba9frFj0xb9qqpG0dXdb9aspeI8k8fiI+fsY=rqGqVepae9pg0db9vqaiVgFr0xfr=xfr=xc9adbaqaaeGacaGaaiaabeqaaeqabiWaaaGcbaGaemyta00aa0baaSqaaiabdMgaPbqaaiabdogaJbaakiabcIcaOGGaciab=f7aHjabcMcaPaaa@332F@:

log⁡2(Ci(α))=log⁡(Mic(α))−log⁡(Mi1(α))c1+(c2−1)log⁡(Mi1(α)),
 MathType@MTEF@5@5@+=feaafiart1ev1aaatCvAUfKttLearuWrP9MDH5MBPbIqV92AaeXatLxBI9gBaebbnrfifHhDYfgasaacPC6xNi=xI8qiVKYPFjYdHaVhbbf9v8qqaqFr0xc9vqFj0dXdbba91qpepeI8k8fiI+fsY=rqGqVepae9pg0db9vqaiVgFr0xfr=xfr=xc9adbaqaaeGacaGaaiaabeqaaeqabiWaaaGcbaGagiiBaWMaei4Ba8Maei4zaC2aaSbaaSqaaiabikdaYaqabaGccqGGOaakcqWGdbWqdaWgaaWcbaGaemyAaKgabeaakiabcIcaOGGaciab=f7aHjabcMcaPiabcMcaPiabg2da9KqbaoaalaaabaGagiiBaWMaei4Ba8Maei4zaCMaeiikaGIaemyta00aa0baaeaacqWGPbqAaeaacqWGJbWyaaGaeiikaGIae8xSdeMaeiykaKIaeiykaKIaeyOeI0IagiiBaWMaei4Ba8Maei4zaCMaeiikaGIaemyta00aa0baaeaacqWGPbqAaeaacqaIXaqmaaGaeiikaGIae8xSdeMaeiykaKIaeiykaKcabaGaem4yam2aaSbaaeaacqaIXaqmaeqaaiabgUcaRiabcIcaOiabdogaJnaaBaaabaGaeGOmaidabeaacqGHsislcqaIXaqmcqGGPaqkcyGGSbaBcqGGVbWBcqGGNbWzcqGGOaakcqWGnbqtdaqhaaqaaiabdMgaPbqaaiabigdaXaaacqGGOaakcqWFXoqycqGGPaqkcqGGPaqkaaGaeiilaWcaaa@6C18@

*C*_*i*_(*α*) > 0. This equation remains true if *C*_*i*_(*α*) is not an integer.

As we have only *I*_*i*_(*α*), an estimate of Mic(α)
 MathType@MTEF@5@5@+=feaafiart1ev1aaatCvAUfKttLearuWrP9MDH5MBPbIqV92AaeXatLxBI9gBaebbnrfifHhDYfgasaacPC6xNi=xH8viVGI8Gi=hEeeu0xXdbba9frFj0xb9qqpG0dXdb9aspeI8k8fiI+fsY=rqGqVepae9pg0db9vqaiVgFr0xfr=xfr=xc9adbaqaaeGacaGaaiaabeqaaeqabiWaaaGcbaGaemyta00aa0baaSqaaiabdMgaPbqaaiabdogaJbaakiabcIcaOGGaciab=f7aHjabcMcaPaaa@332F@, we can only obtain Xic(α)
 MathType@MTEF@5@5@+=feaafiart1ev1aaatCvAUfKttLearuWrP9MDH5MBPbIqV92AaeXatLxBI9gBaebbnrfifHhDYfgasaacPC6xNi=xH8viVGI8Gi=hEeeu0xXdbba9frFj0xb9qqpG0dXdb9aspeI8k8fiI+fsY=rqGqVepae9pg0db9vqaiVgFr0xfr=xfr=xc9adbaqaaeGacaGaaiaabeqaaeqabiWaaaGcbaGaemiwaG1aa0baaSqaaiabdMgaPbqaaiabdogaJbaakiabcIcaOGGaciab=f7aHjabcMcaPaaa@3345@, an estimate of log_2_(*C*_*i*_(*α*)). We assume that Xic(α)
 MathType@MTEF@5@5@+=feaafiart1ev1aaatCvAUfKttLearuWrP9MDH5MBPbIqV92AaeXatLxBI9gBaebbnrfifHhDYfgasaacPC6xNi=xH8viVGI8Gi=hEeeu0xXdbba9frFj0xb9qqpG0dXdb9aspeI8k8fiI+fsY=rqGqVepae9pg0db9vqaiVgFr0xfr=xfr=xc9adbaqaaeGacaGaaiaabeqaaeqabiWaaaGcbaGaemiwaG1aa0baaSqaaiabdMgaPbqaaiabdogaJbaakiabcIcaOGGaciab=f7aHjabcMcaPaaa@3345@ is normally distributed around log_2_(*C*_*i*_(*α*)) with standard deviation *σ*_*c*_.

The parameters Mi1(α)
 MathType@MTEF@5@5@+=feaafiart1ev1aaatCvAUfKttLearuWrP9MDH5MBPbIqV92AaeXatLxBI9gBaebbnrfifHhDYfgasaacPC6xNi=xH8viVGI8Gi=hEeeu0xXdbba9frFj0xb9qqpG0dXdb9aspeI8k8fiI+fsY=rqGqVepae9pg0db9vqaiVgFr0xfr=xfr=xc9adbaqaaeGacaGaaiaabeqaaeqabiWaaaGcbaGaemyta00aa0baaSqaaiabdMgaPbqaaiabigdaXaaakiabcIcaOGGaciab=f7aHjabcMcaPaaa@32D0@, *c*_1_, *c*_2_, *σ*_1_, *σ*_2 _were estimated using the HapMap data set based on the knowledge of the allelic copy-numbers of each SNP; i.e. 0, 1, or 2 depending on whether the SNP is heterozygous or homozygous. Here *c*_1 _= -0.38, *c*_2 _= 1.08, *σ*_1 _= 0.3 and *σ*_2 _= 0.35. We assume that *σ*_*c *_= *σ*_2 _for *c *> 2. When the copy-number is 0, we can still use equation 4 with Xi0(α)
 MathType@MTEF@5@5@+=feaafiart1ev1aaatCvAUfKttLearuWrP9MDH5MBPbIqV92AaeXatLxBI9gBaebbnrfifHhDYfgasaacPC6xNi=xH8viVGI8Gi=hEeeu0xXdbba9frFj0xb9qqpG0dXdb9aspeI8k8fiI+fsY=rqGqVepae9pg0db9vqaiVgFr0xfr=xfr=xc9adbaqaaeGacaGaaiaabeqaaeqabiWaaaGcbaGaemiwaG1aa0baaSqaaiabdMgaPbqaaiabicdaWaaakiabcIcaOGGaciab=f7aHjabcMcaPaaa@32E4@ being distributed as a normal distribution with mean -2 and *σ*_0 _= 0.55 (empirical observation; see additional file 2). A level of -2 corresponds to 0.25 copies, and not to 0 copies. This slightly elevated level can be explained by cross-hybridization and background noise. Those values were also obtained using the HapMap data set.

### Selection of SNPs

We selected only the SNPs that conformed well to the model. Those that do not conform to the model are less likely to be useful for copy-number analysis [8]. The selection is based on the normal samples from each group: the 90 HapMap samples, the 38 normal samples from the prostate group and the 29 normal samples from the bladder group. A SNP is selected if it has a high call rate (above 90%) (Affymetrix genotype call) and if there is a high correspondence between the inferred allelic copy-number given by equation 4 and the true allelic copy-number given by the genotype (see [8] for more details).

### A Hidden Markov Model (HMM) to estimate the allelic copy-number

#### The model

We used a HMM to estimate the allelic copy-number of the selected SNPs. Our HMM has six states (Figures 1A, 1B) corresponding to the germline state (state 0) and five chromosomal abnormalities: heterozygous deletion (state 1), homozygous deletion (state 2), uniparental di/polysomy (state 3), unbalanced amplification (state 4) and balanced amplification (state 5).

We defined the transition matrix using 3 parameters (Figure 1c). The transition probabilities are the probabilities of moving from one state to another state, when moving from one SNP to its neighbour SNP. The *p*-parameter is the probability of moving from the germline state (state 0) to an abnormal state (states 1 to 5). The *r*-parameter is the probability of moving from one abnormal state to another different abnormal state and the *ε*-parameter is the probability of what is considered an improbable transition (Figure 1d). We considered a transition improbable if the transition implies two breakpoints between two consecutive SNPs. For example, the transition between the germline state (state 0) and the uniparental di/polysomy state (state 3) is improbable as it implies one breakpoint to lose one allele and a second breakpoint to multiplicate the other allele.

For each state, the emission density is defined as a bivariate normal distribution where the mean is the logarithm of the allelic copy-numbers and the covariance matrix (including *σ*_*c*_) is estimated from the normal samples. For the states with copy-numbers 2+, 3+ or 4+, we take the mean to be the logarithm of 2, 3 or 4 copies, respectively. Looking at the HapMap data set, we could estimate the mean of Xi0
 MathType@MTEF@5@5@+=feaafiart1ev1aaatCvAUfKttLearuWrP9MDH5MBPbIqV92AaeXatLxBI9gBaebbnrfifHhDYfgasaacPC6xNi=xH8viVGI8Gi=hEeeu0xXdbba9frFj0xb9qqpG0dXdb9aspeI8k8fiI+fsY=rqGqVepae9pg0db9vqaiVgFr0xfr=xfr=xc9adbaqaaeGacaGaaiaabeqaaeqabiWaaaGcbaGaemiwaG1aa0baaSqaaiabdMgaPbqaaiabicdaWaaaaaa@2F82@. When a SNP marker was homozygous in the germline DNA, we defined the emission densities as a normal density as only one allele could be present in the cancer cells.

The Viterbi algorithm is used to recover the hidden states and a modified version of the Baum-Welch algorithm is used to estimate the *p *and *r*-parameters. The *ε*-parameter is set to an arbitrary but small value. Here *ε *= 0.00001.

#### Simulation of data sets

In order to test if the method can recover known transition parameters and known states, we simulated data sets with different transition parameters. For the simulation, we used 18 arrays from the international HapMap project in order to estimate the noise corresponding to 0, 1 or 2 copies of an allele. The noise was defined as the difference between the observed log copy-number Xic(α)
 MathType@MTEF@5@5@+=feaafiart1ev1aaatCvAUfKttLearuWrP9MDH5MBPbIqV92AaeXatLxBI9gBaebbnrfifHhDYfgasaacPC6xNi=xH8viVGI8Gi=hEeeu0xXdbba9frFj0xb9qqpG0dXdb9aspeI8k8fiI+fsY=rqGqVepae9pg0db9vqaiVgFr0xfr=xfr=xc9adbaqaaeGacaGaaiaabeqaaeqabiWaaaGcbaGaemiwaG1aa0baaSqaaiabdMgaPbqaaiabdogaJbaakiabcIcaOGGaciab=f7aHjabcMcaPaaa@3345@ and the true log copy-number, log_2_(*C*_*i*_(*α*)). To estimate the noise corresponding to 0 copies, we used a log copy-number equal to -2; as described previously. Then, we used one HapMap sample and replaced the normalized intensity for each SNP and allele by a simulated value corresponding to a known state with noise estimated from the HapMap sample. The states were determined randomly using the HMM model. Here, all the SNPs were given a heterozygous call.

Further, we simulated a mixture of cancer and normal cells. Here we determined the observed values by adding noise obtained from the Hapmap data set to the allelic copy-number defined as follows:

*C*_*O *_= (1 - *m*)*C*_*N *_+ *mC*_*T *_

where *C*_*O *_is the allelic copy-number (i.e. the average copy-number in the mixture population), *C*_*N *_and *C*_*T *_are the allelic copy-numbers in the normal and the cancer cells and *m *is the percentage of cancer cells present in the mixture.

### Including the mixture proportion

#### The mixture model

We modified the HMM defined above in order to account for a mixture of normal and cancer cells. This was done in the emission probabilities where the allelic copy-number was considered a weighted sum of the copy-numbers from the normal and the abnormal cells (see equation 5). Then we used an iterative procedure to estimate the mixture proportion, *m*, in a sample (*m *is the proportion of cancer cells).

• Initialisation: we ran the method on the sample considering there is no mixture (*m *= 100%) and obtained a sequence of hidden states corresponding to the sample.

• Update 1: Assuming the sequence of hidden states, we used a least-square method to fit the optimal mixture value *m*.

• Update 2: Assuming *m*, we applied the method with the mean intensity given as in equation 5. A new sequence of hidden states was obtained.

• Iteration step: We repeated Update 1 and Update 2 until the mixture *m *did not change.

As only SNPs (or alleles) in abnormal states can help in obtaining an estimate of the mixture level, we need to have a minimum of changes in copy-numbers occurring in order to obtain a reasonable estimate. The iterative procedure was applied only to samples containing more than 5% of abnormal states after the initialisation.

#### Simulation of data sets

In order to test the iterative procedure on more realistic simulated samples, we designed the simulations in a different way. We first ran the HMM on the real bladder and prostate tumour samples, then we used the sequence of hidden states recovered to produce new simulated samples with known population mixture. The observed allelic copy-numbers were determined as in equation 5.

### The all-array method

Until now, the transition parameters were estimated for each sample but were the same for all chromosomes. Here, we modified the method to allow different transition parameters for each chromosome.

#### Simulation of data sets

We simulated two data sets of 20 samples where the transition parameters were randomly chosen between 0.001 and 0.05 on the logarithmic scale. For each of the 40 samples, a sequence of hidden states was determined according to the HMM. Each chromosome had its own transition parameters across all samples. In the first data set of 20 samples, only heterozygous SNP intensities were simulated. Each sample had the same number of SNPs and the same positions in the genome as the HapMap samples. In the second data set of 20 samples, each sample had the same number of SNPs, the same positions in the genome and the same genotype calls as one randomly chosen HapMap sample. SNP intensities were simulated according to the genotype call and to the hidden state determined previously for this SNP.

## Authors' contributions

PL, CLA and CW designed the study; PL wrote the paper, implemented the method and performed the analysis. CLA and CW commented on the manuscript. LD provided the bladder data and NT the prostate data. All authors read and approved the final manuscript.

## Supplementary Material

Additional File 1 Linear relationship between mean intensities. The figure shows the average intensities for 1 copy of an allele (blue) and the average intensities for 2 copies of an allele (blue) plotted against the estimated mean intensities for 1 copy, using the model described in [8]. The parameter *c*1 is the intercept of the top line and *c*_2 _is the slope, see equation 2. The slope of the bottom line is 1 and the intercept 0. Click here for file

Additional File 2 Histograms of allelic intensities for the HapMap data. The figure shows the histograms of the normalized intensities corresponding to 0, 1 or 2 copies. Click here for file
