# Supplementary figures and images for: A Hidden Markov Model to estimate population mixture and allelic copy-numbers in cancers using Affymetrix SNP arrays
Source: BMC Bioinformatics. 2007 Nov 9;8:434. doi: 10.1186/1471-2105-8-434 (PMC2206057; doi:10.1186/1471-2105-8-434)

## Linear relationship between mean intensities

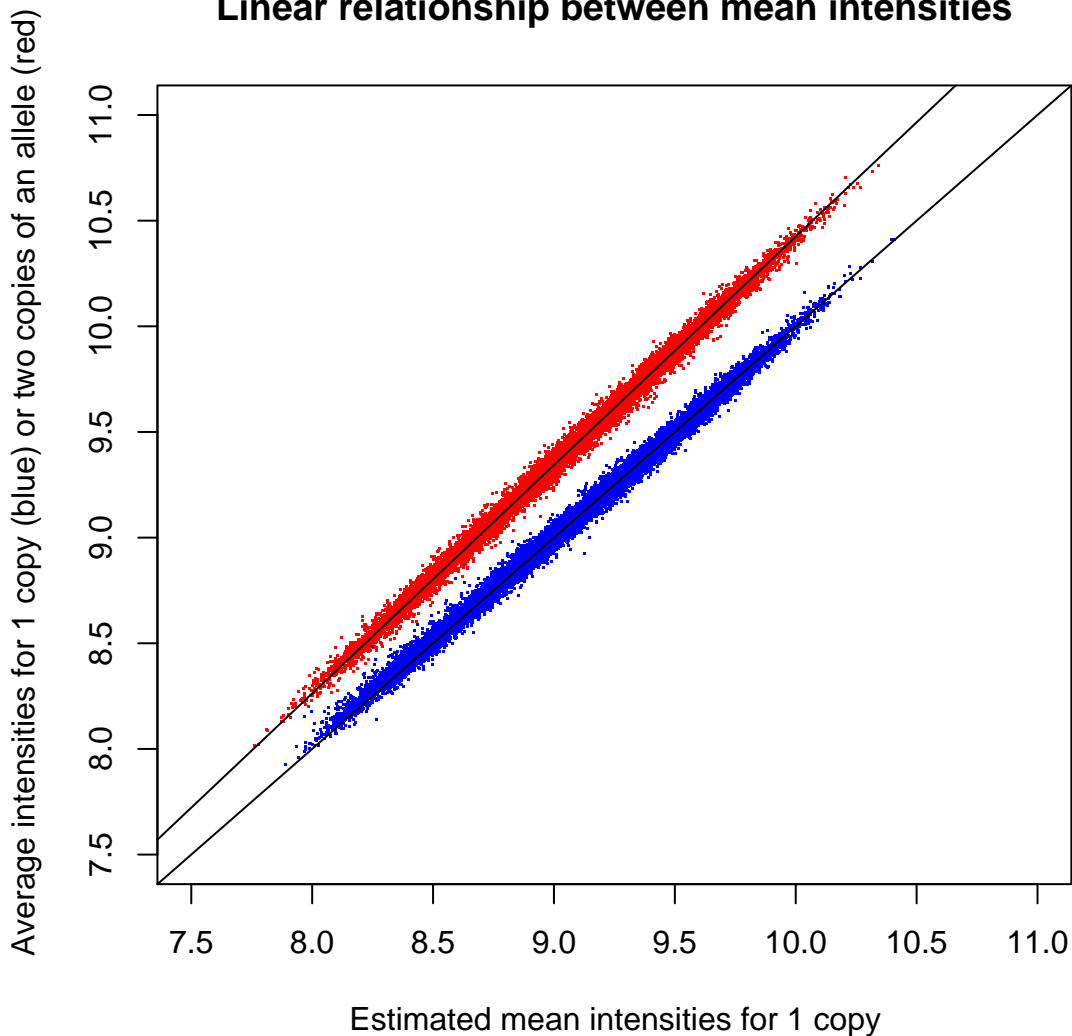

Supplement: Additional File 1 — Linear relationship between mean intensities. The figure shows the average intensities for 1 copy of an allele (blue) and the average intensities for 2 copies of an allele (blue) plotted against the estimated mean intensities for 1 copy, using the model described in [8]. The parameter c1 is the intercept of the top line and c2 is the slope, see equation 2. The slope of the bottom line is 1 and the intercept 0. [file 1471-2105-8-434-S1.pdf]
