# Supplementary material for: A Hidden Markov Model to estimate population mixture and allelic copy-numbers in cancers using Affymetrix SNP arrays
Source: BMC Bioinformatics. 2007 Nov 9;8:434. doi: 10.1186/1471-2105-8-434 (PMC2206057; doi:10.1186/1471-2105-8-434)
Supplement: Additional File 2 — Histograms of allelic intensities for the HapMap data. The figure shows the histograms of the normalized intensities corresponding to 0, 1 or 2 copies. [file 1471-2105-8-434-S2.pdf]

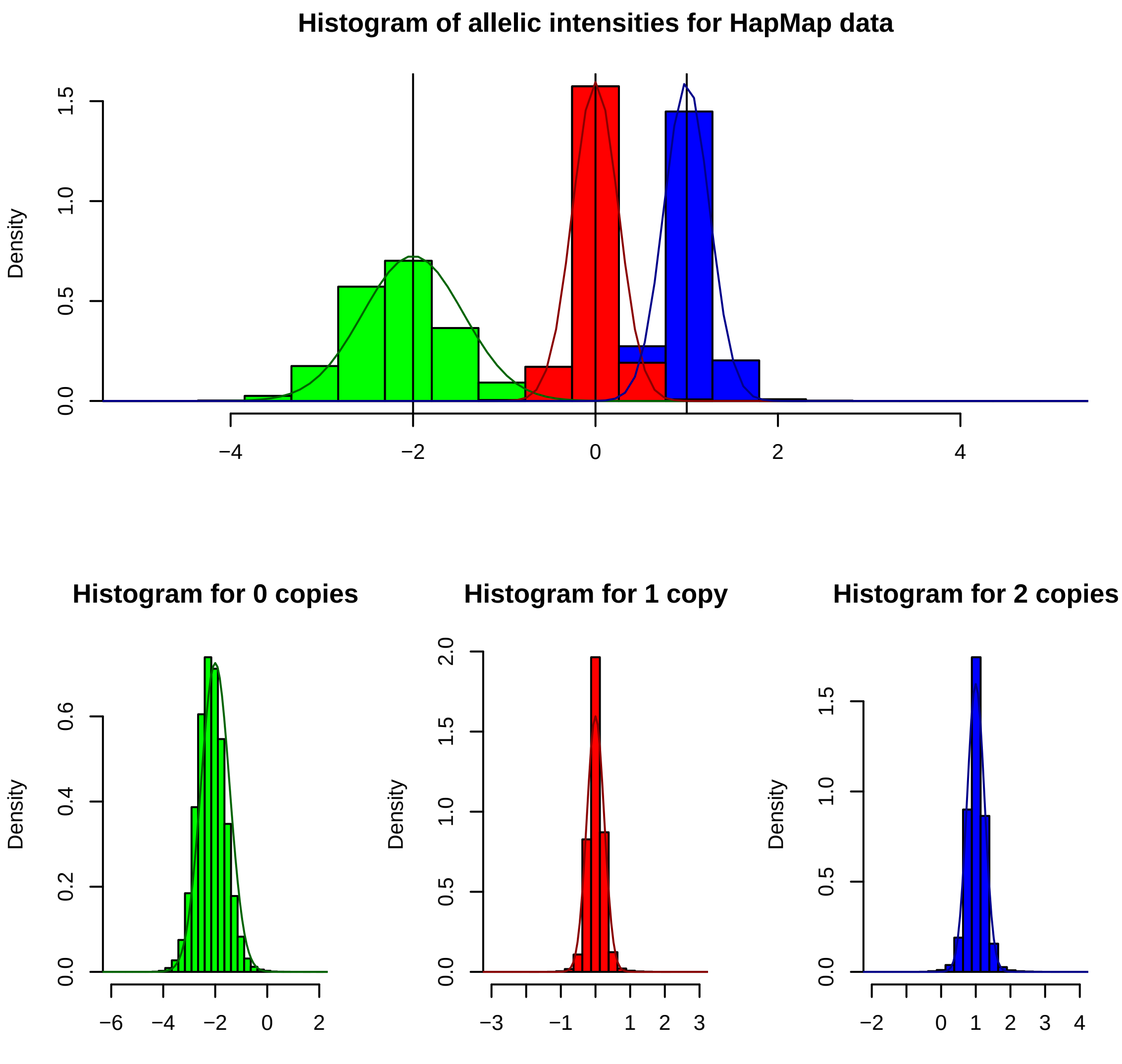
Histogram of allelic intensities for HapMap data
Density
0.0
0.5
1.0
1.5
−4
−2
0
2
4
Histogram for 0 copies
Density
0.0
0.2
0.4
0.6
−6
−4
−2
0
2
Histogram for 1 copy
Density
0.0
0.5
1.0
1.5
2.0
−3
−1
1
2
3
Histogram for 2 copies
Density
0.0
0.5
1.0
1.5
−2
0
1
2
3
4
